# Allopregnanolone Concentrations After Ascending Single Dose Administration of Progesterone to Healthy Volunteers

**DOI:** 10.1002/hup.70012

**Published:** 2025-07-23

**Authors:** Natalie J. Hughes‐Medlicott, Hoang Nguyen, Paul Glue, Yoram Barak

**Affiliations:** ^1^ School of Pharmacy Otago University Dunedin New Zealand; ^2^ Department of Psychological Medicine Dunedin School of Medicine Otago University Dunedin New Zealand

**Keywords:** allopregnanolone, pharmacokinetics, progesterone

## Abstract

**Background:**

Postpartum depression (PPD) is associated with significant morbidity and mortality. It affects as many as 11.5% of women giving birth. Allopregnanolone (an endogenous progesterone metabolite) has been a promising avenue of clinical research for the treatment of PPD.

**Aim:**

To assess the pharmacokinetics of allopregnanolone (Allo) following orally dosed progesterone in healthy volunteers. Secondary outcome was calculating the daily dose of progesterone needed to achieve the clinically meaningful concentration of 50 ng/mL Allo.

**Methods:**

Single ascending dose study to measure plasma concentrations of Allo after 200, 400 and 600 mg doses of extended‐release progesterone capsules. Secondary outcome was the safety and tolerability of extended‐release progesterone capsules.

**Results:**

We recruited 10 participants, 9 male and 1 female, mean (SD) age 38.7 (18.7) years. The maximum plasma concentration (*C*_max_) of Allo was observed at 2 h. A linear relationship was fitted to the observations. Sedation was assessed at baseline, 1, 2, 4, 6 and 8 h after each dose. Sedation ratings increased at 1–2 h post‐dose after all three progesterone doses, with the greatest increase after the 600 mg dose, and fell subsequently. Vital signs were unchanged, and no other adverse events were reported.

**Conclusions:**

In this single ascending dose study has clarified that 400 mg four times/day of progesterone is required to achieve maximum plasma ALLO concentrations of 50 ng/mL. Tolerability and safety were acceptable for all doses of progesterone tested.

## 1 Introduction

Six years ago, brexanolone, a neuroactive steroid *γ*‐aminobutyric acid receptor‐positive allosteric modulator, was approved by the United States Food and Drug Administration (FDA) as the first‐ever drug specifically for postpartum depression (Meltzer‐Brody et al. 2018). Postpartum depression (PPD) may be provoked by GABA‐A receptors' failure to adapt to abrupt reductions in allopregnanolone (Allo; 3α, 5α‐tetrahydroprogesterone) levels after childbirth (Kanes et al. 2017). Publication of the results of two double‐blind, randomised, placebo‐controlled, phase III trials again demonstrated positive outcome for intravenous brexanolone in PPD (Meltzer‐Brody et al. 2018). Intense research and clinical efforts followed leading to designing and testing an oral neuroactive steroid *γ*‐aminobutyric acid receptor‐positive allosteric modulator, in PPD, named zuranolone. In a Phase 3 randomized controlled trial enrolling 153 women across the USA zuranolone improved the core symptoms of depression in women with PPD, was well tolerated, supporting further development of oral neuroactive steroid modulation in the treatment of PPD (Deligiannidis et al. 2021).

We recently hypothesized that repeated doses of an oral formulation of progesterone could be used as an alternative to brexanolone or zuranolone (Barak and Glue 2020). Progesterone is metabolized in vivo to Allo. In animal models, progesterone prevents depression‐like behaviours possibly acting as a neuroprotective agent for both motor deficits as well as cognitive, memory, and depression‐like behaviours (Casas et al. 2011; Frye and Walf 2009; Li et al. 2013). Two recent publications partly support the positive effects of progesterone‐containing contraceptives on the rates of PPD or depression in the first 12 months postpartum (Ti and Curtis 2019; Roberts and Hansen 2017).

We are concerned that the proposed cost of brexanolone at US$34,000 (Post) and the estimated cost of zuranolone 30 mg for 14 days of treatment (US$5600 (Selleck 2022)) will impact on the use and availability of what appears to be an important new therapeutic for PPD. In 2023, the FDA granted marketing authorization for zuranolone for postpartum depression. However, there are several features of the design and conduct of the drug's pivotal trials that undermine confidence in this agent, which should give physicians pause in prescribing (Prasad and Allely 2024).

On the other hand, progesterone capsules cost NZ$0.55 each, that is US$0.39 (Pharmac 2019). Oral progesterone for PPD would be inexpensive, easy to administer, and could facilitate wider use of this important new treatment. This may further lead to exploring additional alternative endocrine‐replacement treatments for PPD (Ahokas et al. 2000).

The aim of the present study was to explore whether plasma Allo concentrations could be increased via oral progesterone loading.

## 2 Methods

This study was approved by the New Zealand Health and Disability Ethics Committees; ethics reference: 20/CEN/205/AM02. Participants gave their signed informed consent after reading a detailed structured safety data, information sheet and a consent form.

### 2.1 Design

This was an ascending dose study, to measure plasma Allo concentrations after single doses of 200 mg, 400 mg and 600 mg of extended‐release progesterone capsules (Utrogestan 200 mg capsules). Doses were separated by at least on week. Secondary outcomes were the safety and tolerability of extended‐release progesterone capsules in healthy volunteers. We recruited healthy male and post‐menopausal females for this study. Inclusion criteria included being in good general health, aged 20–60, weighing at least 50 kg, with a minimum BMI of 18. Exclusion criteria included having severe or unstable medical conditions, and regular use of alcohol and or recreational drugs.

Plasma samples to measure Allo concentrations were obtained pre‐dose and 2‐, 4‐, 6‐ and 8‐h post‐dose. Plasma samples were assayed using an Allo competitive ELISA method (Invitrogen, ThermoFisher Scientific, New Zealand). Plasma concentrations were analysed by non‐compartmental pharmacokinetic methods using GraphPad Prism (Version 9.5.1). The primary endpoint was peak Allo concentrations, along with an assessment of dose‐proportionality. We calculated the total dose to achieve a Cmax of 50 mg/mL assuming that progesterone has linear pharmacokinetics, and through extrapolation of the dose/Cmax relationship. Safety and tolerability were assessed by reported adverse events and vital signs throughout the study. Self‐ratings of sedation were obtained using a 100 mm visual analogue scale, where 0 represented ‘not at all sleepy’, and 100 ‘the sleepiest ever’.

## 3 Results

We recruited 10 participants, 9 male and 1 female, mean (SD) age 38.7 (18.7) years. All participant were white.

**FIGURE 1:**
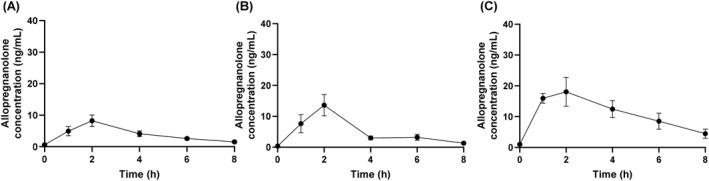
Mean (SEM) allopregnanolone concentrations following oral doses of (A) 200 mg (*n* = 10), (B) 400 mg (*n* = 7) and (C) 600 mg (*n* = 6) progesterone.

Plasma concentrations of Allo are shown in Figure 1.

**FIGURE 2:**
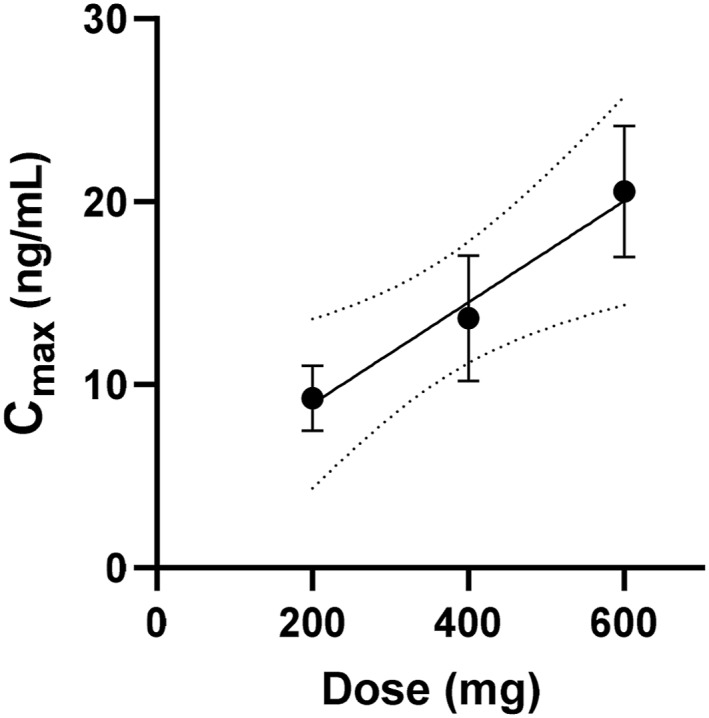
Maximum plasma concentrations of allo following oral administration of 200, 400 and 600 mg progesterone. Error bars represent the standard error of the mean (*n* = 10, 7 and 6 respectively) and 95% confidence intervals are shown as dotted lines.

The maximum plasma concentration (*C*_max_) of Allo was observed at 2 h. A linear relationship was fitted to the observations (*n* = 23) which gave a slope of 0.0277 ± 0.0095 and *y*‐intercept of 3.4 ± 3.8 with an *R* ^2^ of 0.29 (Figure 2).

**FIGURE 3:**
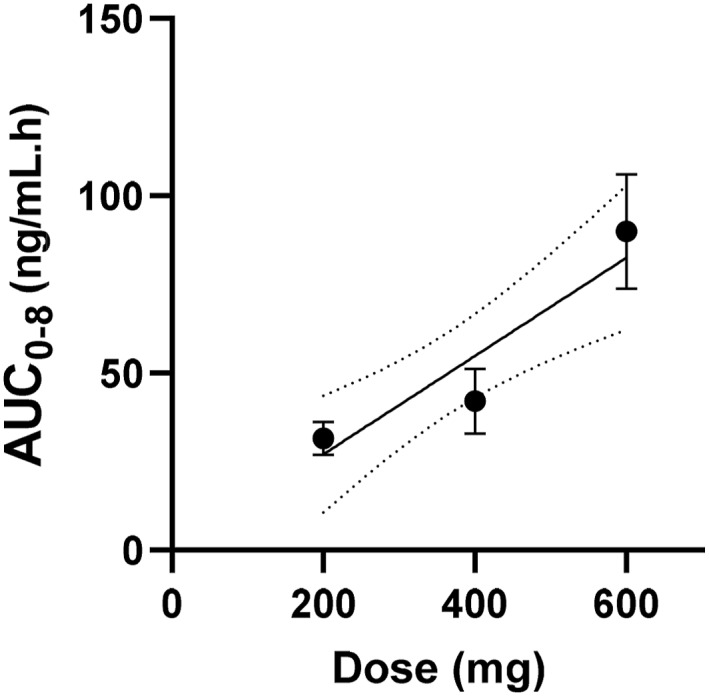
Area under the curve (AUC) from 0 to 8 h for Allo following oral administration of 200, 400 and 600 mg progesterone. Error bars represent the standard error of the mean (*n* = 10, 7 and 6 respectively) and 95% confidence intervals are shown as dotted lines.

The mean area under the plasma concentration—time curves (AUC_0 to 8_) hours is shown in Figure 3. Again, least squares linear regression was carried out using individual observations. The slope was 0.139 ± 0.034 and intercept was 4.03 ± 13.6 (*R* ^2^ = 0.439).

To estimate what daily dose of progesterone would be needed to achieve an Allo Cmax of 50 ng/mL, if we assumed linear pharmacokinetics and extrapolated the linear regression of Cmax versus dose from Figure 2. This gave an estimated a total daily dose of progesterone of 1680 mg.

**FIGURE 4:**
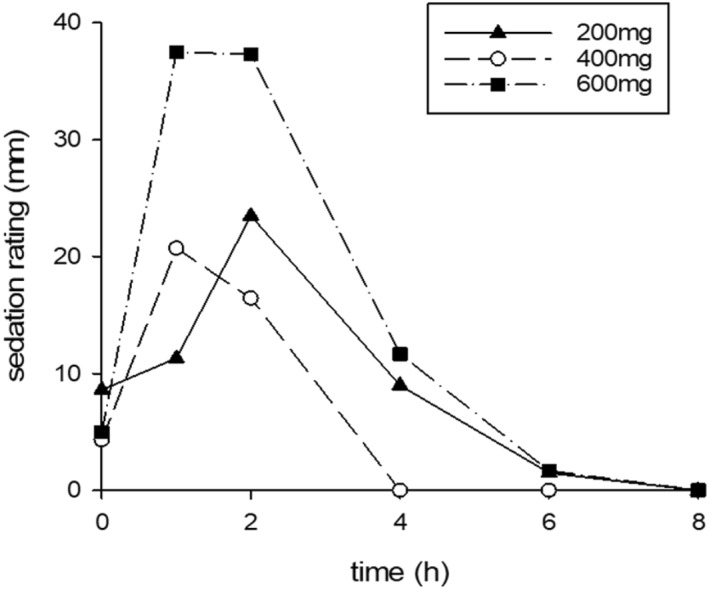
Effect of progesterone 200, 400 and 600 mg on sedation self‐ratings.

Safety and tolerability: Sedation was assessed at baseline, 1, 2, 4, 6 and 8 h after each dose using a 100 mm visual analogue scale, Sedation ratings increased at 1–2 h post‐dose after all three progesterone doses, with the greatest increase after the 600 mg dose, and fell subsequently (Figure 4). Vital signs were unchanged, and no other adverse events were reported.

## 4 Discussion

In the past year, 68 clinical trials focusing on the treatment of PPD were published. Of those, the minority of interventions were pharmacological, with only oxytocin, ketamine and zuranolone being studied. The promising new horizon opened by neurosteroid‐based treatment for depressive disorders and particularly for PPD was emphasized in published reviews (Pinna et al. 2022). The present study aimed to measure plasma Allo concentrations after single ascending doses of extended‐release progesterone capsules. This was conceptualized as a preliminary step in investigating the use of repeat dose progesterone oral loading for PPD (Barak and Glue 2020). Serum Allo concentrations at the end of pregnancy are approximately 50 ng/mL (Luisi et al. 2000), and this was the target concentration attained during maximal dosing of brexanolone (Kanes et al. 2017).

If linear pharmacokinetics is assumed and the linear relationship observed between dose and Cmax shown in Figure 2 was extended then we estimate a total dose of 1680 mg could achieve a Cmax of 50 ng/mL. However, this prediction has relatively wide confidence limits, due to the variability in concentrations and Cmax values achieved in participants in this study.

The present study demonstrated that to achieve a multiple dose Allo Cmax of 50 ng/mL, a dose of 1680 mg oral progesterone would be needed, as progesterone shows linear pharmacokinetics at higher doses. Because brexanolone is not dosed consistently, but is uptitrated on Day 1 of dosing, is dosed maximally on Day 2, and is down‐titrated on Day 3 (Meltzer‐Brody et al. 2018; Kanes et al. 2017), we predict that repeated doses of 400 mg of progesterone given every 6 h on Day 2 would achieve 50 ng/mL Allo concentrations, at a total cost of $NZ15.95 or US$9.54.

The main side effect of single dose progesterone was sedation, which showed dose‐related trends. Peak sedation ratings occurred around the time of peak Allo concentrations. Whether tolerance develops to this after repeated doses of progesterone is unknown.

Limitations of this study include the small number of participants, and the relatively brief duration of pharmacokinetic sampling. The gradual reduction in the number of participants completing each dosing section was due to scheduling difficulties thus failure to continue was not a consequence of any issue with the medication. However, we believe the design was fit for purpose, to provide data to assist in the design of a multiple dose study.

In conclusion, this single ascending dose study has clarified what daily dose of progesterone is required to achieve plasma Allo peak concentrations of 50 ng/mL. Our next study will evaluate the pharmacokinetics of this dosing regimen, along with its safety and tolerability. These data would support future inexpensive trials of oral progesterone in patients with PPD.

## Conflicts of Interest

Drs Hughes Medlicott and Glue are named on a patent for an extended release ketamine formulation. The other authors report no conflict of interests.

## Data Availability

The data that support the findings of this study are available on request from the corresponding author. The data are not publicly available due to privacy or ethical restrictions.
